# Efficacy of krill oil versus fish oil on obesity-related parameters and lipid gene expression in rats: randomized controlled study

**DOI:** 10.7717/peerj.12009

**Published:** 2021-09-27

**Authors:** Mevra Aydin Cil, Atena Ghosi Ghareaghaji, Yasin Bayir, Zehra Buyuktuncer, Halit Tanju Besler

**Affiliations:** 1 Department of Nutrition and Dietetics, Faculty of Health Sciences, Hacettepe University, Ankara, Turkey; 2 Department of Nutrition and Dietetics, Faculty of Health Sciences, Atatürk University, Erzurum, Turkey; 3 Department of Molecular Biology and Genetics, Faculty of Science, Atatürk University, Erzurum, Turkey; 4 Department of Biochemistry, Faculty of Pharmacy, Atatürk University, Erzurum, Turkey; 5 Department of Nutrition and Dietetics, Faculty of Health Sciences, Istinye University, Istanbul, Turkey

**Keywords:** Krill oil, Fish oil, Obesity, LC n-3 PUFA, Fatty acid desaturase, Gene expression

## Abstract

**Backround:**

This study aimed to determine the effects of LC n-3 PUFA supplementation on the prevention and treatment of obesity and obesity-related diseases, and to compare the efficiency of different LC n-3 PUFA sources via biochemical and genetic mechanisms in rats.

**Methods:**

Male Wistar rats were randomized into four study groups, and fed with a standard diet, High Fat Diet (HFD), HFD+%2.5 Fish Oil (FO-HFD) or HFD+%2.5 Krill Oil (KO-HFD) for eight weeks. Food consumption, weight gain, serum glucose, insulin, ghrelin and leptin concentrations, lipid profile, liver fatty acid composition, and FADS1 and FADS2 mRNA gene expression levels were measured.

**Results:**

Weight gain in each HFD group was significantly higher than control group (*p* < 0.001), without any differences among them (*p* < 0.05). LC n-3 PUFAs modified lipid profile, but not glucose tolerance. Serum leptin levels were significantly higher in HFD groups than in the control group, however, no difference in serum ghrelin levels was observed among the groups. Liver n-3 fatty acid desaturation activity was higher (*p* = 0.74), and liver total lipid content was lower (*p* = 0.86) in KO-HFD compared to FO-HFD. FADS1 gene expression was highest in the HFD group (*p* < 0.001) while FADS2 gene expression was highest in the FO-HFD group (*p* < 0.001).

**Conclusion:**

LC n-3 PUFAs, especially krill oil, had moderate effects on lipid profile, but limited effects on obesity related parameters, suggesting different effects of different sources on gene expression levels. Further randomized controlled trials are needed to determine the efficacy of different LC n-3 PUFA sources in the prevention and treatment of obesity in humans.

## Introducion

Obesity is a complex and multifactorial chronic disease, which is also recognized as a global public health problem due to the increasing worldwide prevalence (Bender et al., 2014; Gregor & Hotamisligil, 2011). World Health Organization (WHO) 2016 data showed that approximately 1.9 billion adults (39%) are overweight, and 650 million adults (13%) are obese (World Health Organization, 2020). In addition to its high prevalence, obesity is also a significant risk factor for non-communicable chronic diseases such as type 2 diabetes (T2DM), cardiovascular diseases (CVD), non-alcoholic fatty liver disease (NAFLD) and various cancers, leading to further morbidity and mortality (Jung & Choi, 2014; World Health Organization, 2020). Obesity and its comorbidities represent a state of low-grade inflammation that can cause the dysfunction of the specific areas in the hypothalamus where the feeding behavior and energy expenditure are regulated (Gregor & Hotamisligil, 2011).

Long chain n-3 polyunsaturated fatty acids (LC n-3 PUFAs) have been suggested as potential functional food components against obesity due to their various biological activities. First, LC n-3 PUFAs may have beneficial effects on low-grade inflammation in obesity *via* their anti-inflammatory activity (Buckley & Howe, 2010; Campioli, Rustichelli & Avallone, 2012). Second, LC n-3 PUFAs may potentially suppress appetite and food consumption by regulating the hormones related to appetite such as leptin and ghrelin (Bargut, Mandarim-de Lacerda & Aguila, 2015; Parra et al., 2008; Pérez-Matute et al., 2007). Also, LC n-3 PUFAs can tilt orexigenic/anorexigenic balance of the arcuate nucleus towards the anorexigenic side, causing anorexigenic effects by increasing the activity of the ventromedial hypothalamic nucleus/paraventricular hypothalamic nucleus (Huang et al., 2004; Saidpour et al., 2012; Selenscig et al., 2010; Watanabe et al., 2009). Third, LC n-3 PUFAs can regulate gene expression of sterol regulatory element binding protein 1 (SREBP-1) and peroxisome proliferators activated receptors (PPARs) in liver and skeletal and cardiac muscles, resulting in an increase of lean tissue and a decrease of fat deposition through the stimulation of fat oxidation and energy expenditure (Buckley & Howe, 2010; Tai & Ding, 2010). Fatty acid desaturation is the key metabolic pathway for the synthesis of LC n-3 PUFAs (Wang et al., 2006). Desaturase enzymes including delta-5 desaturase (D5D) and delta-6 desaturase (D6D) are encoded by the fatty acid desaturase 1 (Fatty Acid Desaturase 1-FADS1) and fatty acid desaturase 2 (Fatty Acid Desaturase 2-FADS2) genes, respectively (Zhang, Kothapalli & Brenna, 2016). The expression levels of these genes determine the desaturase enzyme activities that might be associated with insulin resistance and obesity (Warensjö et al., 2009). Furthermore, LC n-3 PUFAs can control the expression levels of these genes (Chisaguano et al., 2013; Reardon et al., 2013; Su et al., 2016).

The major source of LC n-3 PUFAs supplements in the market is fish oil, however, Antarctic krill (Euphausia superba) has been recognized as a rich source of LC n-3 PUFAs. However, fish oil and krill oil have different fatty acid compositions and metabolic effects. First, the krill oil contains less eicosapentaenoic acid (EPA) and docosahexaenoic acid (DHA) compared to fish oil. Second, LC n-3 PUFAs in krill oil are in phospholipid form whereas they are in triglyceride form in fish oil, which explains the increased the bioavailability of LC n-3 PUFAs in krill oil. Finally, krill oil is rich in astaxanthin, a type of carotenoids with a strong antioxidant activity that can protect the LC n-3 PUFAs against oxidation (Cunningham, 2012; Tou, Jaczynski & Chen, 2007). Studies that have investigated the effects of LC n-3 PUFAs from fish oil on obesity provided contradictory results (Bender et al., 2014; Buckley & Howe, 2010; Du et al., 2015; Simopoulos, 2016). Furthermore, the effects of LC n-3 PUFAs from krill oil on obesity or the comparison of the effectiveness between the LC n-3 PUFAs sources on obesity related parameters are yet to be known (Ulven & Holven, 2015). To our knowledge, this is the first report that has examined the effect of krill oil on the appetite hormones including leptin and ghrelin. Accordingly, the primary objective of this study was to examine the effects of fish oil and krill oil supplementation on body weight, dietary intake and biochemical parameters related to obesity. The secondary objective was to assess the impact of different LC n-3 PUFAs sources on fatty acid composition of liver and desaturase gene expressions in a rat model.

## Materials & Methods

### Study design, diet and animals

All animal experiments were carried out in accordance with the Guidelines for the Care and Use of Laboratory Animals, and performed under the license from the Local Ethics Committee of Kobay DHL, Ankara, Turkey (Date: 27.04.2017, Protocol number: 226). A total of 33 male Wistar rats, aged eight to ten weeks and weighing 200 to 250 g, were obtained from the Kobay Experimental Animal Laboratory. Wistar rats were preferred for this study because tight control of dietary manipulation was essential to achieve the study objectives.

The rats were housed in standard cages (3 rats per cage) at a constant room temperature of 21 ± 2 °C, a light/dark cycle of 12/12 h and 45% humidity. The rats had *ad libitum* access to food and water during the intervention period. After one week of acclimatization, the rats were randomly divided into four groups: the first group (C, *n* = 6) was fed with a standard chow diet (Special Diet Services-VRF I (P)), the second group (HFD, *n* = 9) was fed with a high fat diet (Testdiet-58Y1), the third group (FO-HFD, *n* = 9) received HFD supplemented with 2.5 wt % menhaden fish oil (Sigma-Aldrich, catalog no: F8020), and the final group (KO-HFD, *n* = 9) received HFD supplemented with 2.5 wt % krill oil (Aker Biomarine, Oslo, Norway) (Ferramosca, Conte & Zara, 2012b; Tandy et al., 2009). In accordance with the experimental protocol, krill oil and fish oil were administered daily by oral gavage. Diets were provided from Kobay Experimental Animal Laboratory in Turkey. Fish oil contained 10–15 g/100 g EPA, 8–15 g/100 g DHA. The EPA and DHA contents of Krill oil (Superba™ 2) were 12.2 g/100 g and 7.1 g/100 g, respectively. The dose of LC n-3 PUFAs used in this study correspond to the average LC n-3 PUFAs intake in humans living in Western countries (Tandy et al., 2009). The macronutrient composition of diets is given in Table 1. Researchers were not blind to intervention. High fat diets were freeze-stored in vacuumed bags until they were used. The food intake was assessed daily by weighing the unconsumed feed at 08.00–10.00 am, and the body weight of rats were measured weekly at the same time interval of the day. At the end of the eight-week intervention period, the animals were sacrificed and blood and liver samples were collected for biochemical and genetic analyses.

**Table 1:** Energy and macronutrient composition of diets.

	C (*n* = 6)	HFD (*n* = 9)	FO-HFD (*n* = 9)	KO-HFD (*n* = 9)
**Energy (kcal/g)**	3,40	5,10	5,32	5,30
**Protein (g/100 g)**	19,11	23,10	23,10	23,10
**Lipid (g/100 g)**	4,75	34,90	37,40	37,10
*Saturated Fatty Acids (SFAs)*	0,75	13,68	14,39	14,25
*Monounsaturated Fatty Acids (MUFAs)*	1,07	14,00	14,50	14,37
*Polyunsaturated Fatty Acids (PUFAs)*	2,66	5,15	5,93	5,78
*n-6 PUFAs*	2,38	4,76	4,91	4,78
*n-3 PUFAs*	0,28	0,39	1,02	1,00
*EPA*	–	–	0,31	0,30
*DHA*	–	–	0,29	0,18
**Carbohydrate (g/100 g)**	55,32	25,90	25,90	25,90

**Notes.**

Energy and macronutrient composition of diets; control (C, *n* = 6), high fat diet (HFD, *n* = 9), fish oil-high fat diet (FO-HFD, *n* = 9) or krill-oil high fat diet (KO-HFD, *n* = 9). n-6 PUFAs: Long chain n-6 polyunsaturated fatty acids, n-3 PUFAs: Long chain n-3 polyunsaturated fatty acids , EPA: Eicosapentaenoic acid DHA: Docosahexaenoic acid.

### Tissue processing

Rats were fasted over 12 h, and then sacrificed by exsanguination under the ether anesthesia, following cardiac puncture (5–10 ml). Serum was separated by centrifugation (3000–3500 rpm, 10 min) and stored at −80 °C until analysis. The livers of the rats weighing 300–500 mg, were subsequently excised and sampled for lipid analyses, and immediately stored at −80 °C. Also samples weighing 30 mg were placed in RNAlater solution for the analysis of gene expression.

### Biochemical analysis

Serum fasting glucose, triglyceride (TG), total cholesterol (TC), high density lipoprotein-cholesterol (HDL-C) and low density lipoprotein-cholesterol (LDL-C) were measured by the enzymatic colorimetric method (Beckman Autoanalyzer, Beckman Instruments, Palo Alto, CA, USA) using commercially appropriate kits (Noeman, Hamooda & Baalash, 2011). Serum insulin (Sunredbio, China), leptin (Sunredbio, China) and ghrelin (Sunredbio, China) analyses were performed using commercially available enzyme-linked immunosorbent assay kits. Homeostasis Model Assessment of Insulin (HOMA-IR) was calculated using the following formula: [HOMA-IR = (insulin (U/l) * glucose (mg/dl)/405] (Matthews et al., 1985).

### Liver fatty acid composition analysis

The lipids were extracted from liver samples as described by Folch, Lees & Sloane-Stanley (1957). Fatty acid methyl esters (FAMEs) were obtained using the method of Metcalfe, Schmitz & Pelka (1966). The separation, quantification and identification of FAMEs were performed using a capillary column (DB-23, 60 m longitude, 0.25 mm ID and 0.25 µm film) and a flame ionization detector in a gas chromatograph/mass spectrometry system (Agilent 6890). A commercial standard (Supelco 37 component FAME mix, Sigma, USA) was used to identify the FAMEs. LC n-3 PUFAs total desaturation activity, EPA/Linolenic Acid ratio and LC n-6 PUFAs desaturase activity were calculated by the arachidonic acid/linoleic acid ratio (Merino et al., 2011).

### Liver FADS1 and FADS2 gene expressions

Total RNA was isolated from liver with a trizol qiazol lysis reagent (Qiagen, Germantown, MD, USA). The concentration and purification were confirmed by a spectrophotometer (Biotek Epoch, USA) at 260/280 nm. After adjusting the RNA concentration, 8 µl from each sample was prepared for the synthesis of cDNA by a RT2 first strand cDNA synthesis kit (Qiagen). The concentration of cDNA was controlled by the spectrophotometer (Biotek Epoch, Winooski, VT, USA). Quantitative RT-PCR was performed at CFX384 Touch (Bio-Rad, Hercules, CA, USA) using a SYBR-Green master mix (Qiagen, USA) fluorescent staining method coupled to double-stranded DNA (Gunel, 2007; Reardon et al., 2013). The expression levels of the target genes (FADS1 and FADS2) and the housekeeping gene (β-actin) as a reference were analyzed. The primer pairs of FADS1 (NM_053445; Qiagen), FADS2 (NM_031344; Qiagen, USA) and β-actin (NM_031144; Qiagen) were provided by Qiagen (Gunel, 2007; Reardon et al., 2013).

### Statistical analysis

SPSS 22.0 for Windows program was used for statistical analysis. A power analysis conducted prior to the experiment showed that with 6 rats in the control group and 9 rats per intervention group at an alpha  = 0.05 there is an 80% power to detect a difference in body weight among the dietary intervention groups. Data were presented as mean ± standard deviation (SD). Kolmogorov–Smirnov and homogeneity tests were used to assess the distribution of data. One-way ANOVA and the Bonferroni test for post hoc analyses were performed to evaluate statistical differences among groups. A value of *p* < 0.05 was considered as significant. Drawing of figures was done with GraphPad Prism 7.04 program.

## Results

**Figure 1:**
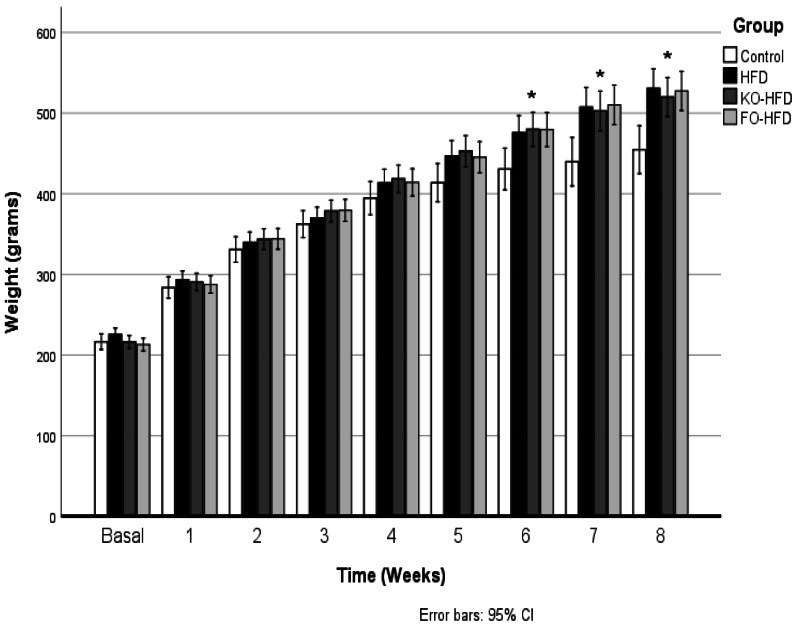
Body weight change during the 8-week of intervention. Control (C, *n* = 6), high fat diet (HFD, *n* = 9), fish oil-high fat diet (FO-HFD, *n* = 9) or krill-oil high fat diet (KO-HFD, *n* = 9). Data is shown as mean. * Weight gain in each study group was significantly higher than control group (*p* < 0.05). *p* was calculated by the one-way ANOVA test, and Bonferroni test was performed for post-hoc analysis.

None of the experimental groups experienced any adverse events, and the study was completed with six rats in control group and nine rats in each intervention group. Figure 1 shows the body weight change during the 8-week intervention period. All the animals in each group gained weight over time, however, body weight change was significantly higher in HFD, FO-HFD and KO-HFD groups compared to control group (*p* < 0.001, for each). Animals fed the FO-HFD had the highest weight gain (%) among the HFD groups (HFD: 135.3 ± 16.7%; FO-HFD: 148.7 ± 13.1% and KO-HFD: 140.7 ±  3.8%), however, only the difference between HFD and FO-HFD groups was statistically significant (*p* = 0.03) (Table 2). The mean feed intake was significantly lower in HFD groups compared to control group (*p* < 0.001). Among the HFD groups, the lowest feed intake was recorded in FO-HFD group (HFD *vs.* FO-HFD *p* = 0.006; FO-HFD *vs.* KO-HFD *p* = 0.01) (Table 2).

**Table 2:** Body weight and feed intake of rats.

	C (*n* = 6)	HFD (*n* = 9)	FO-HFD (*n* = 9)	KO-HFD (*n* = 9)	p
Initial weight^†^	216.5 ± 15.8	225.4 ± 11.4	213.0 ± 9.9	216.2 ± 10.1	0.153
Final weight (g)^†^	454.7 ± 25.0	530.5 ±33.8^a^	527.4 ±49.7^a^	519.8 ±25.2^a^	**0.001**
Weight gain (g)^†^	238.2 ± 15.3	305.1 ±23.24^a^	314.4 ±15.9^a^	303.5 ±40.1^a^	**<0.001**
Weight gain (%)^†^	110.4 ± 9.0	135.3 ± 16.7^a^	148.7 ± 13.1^a,b^	140.7 ± 3.8^a^	**<0.001**
Food intake (g animal^−1^ day^−1^)^‡^	29.4 (29.4–29.5)	18.5^a^ (18.5–18.6)	18.1^a,b^ (16.9–18.5)	18.4^a,c^ (18.4–18.6)	**<0.001**

**Notes.**

Body weight and feed intake of rats fed control (C, *n* = 6), high fat diet (HFD, *n* = 9), fish oil-high fat diet (FO-HFD, *n* = 9) or krill-oil high fat diet (KO-HFD, *n* = 9). Data is shown as mean ± standard deviation or median (minimum-maximum).

a,b,c Mean values with unlike superscript letters were significantly different (*p* < 0.05). ^a^:C *vs.* HFD or C *vs.* FO-HFD or C *vs.* KO-HFD, *p* < 0.05; ^b^: HFD *vs.* FO-HFD, or HFD *vs.* KO-HFD, *p* < 0.05; ^c^: FO-HFD *vs.* KO-HFD, *p* < 0.05.

† *p* was calculated by the One-way Anova test, and Bonferroni test was performed for post-hoc analysis.

‡ *p* was calculated by Kruskall Wallis test, and Mann Whitney U test was performed for post-hoc analysis.

Table 3 summarizes the serum fasting levels of biochemical parameters. Serum TG, TC, LDL-C, glucose and leptin differed significantly among the groups (*p* = 0.002; *p* = 0.001; *p* = 0.02; *p* = 0.02; *p* = 0.03, respectively). HFD group had higher serum concentration of TG, TC and LDL-C than the C group had (*p* = 0.001; *p* = 0.005; *p* = 0.03, respectively). Among the HFD groups, KO-HFD group had the lowest serum TG (HFD *vs.* KO-HFD *p* = 0.04; FO-HFD *vs.* KO-HFD *p* = 0.75) and TC levels (HFD *vs.* KO-HFD *p* = 0.03; FO-HFD *vs.* KO-HFD *p* = 0.02). Both TG and TC were significantly lower in KO-HFD group than the HFD group, while only TC was significantly lower in KO-HFD group than the FO-HFD group (*p* = 0.02). Therefore, krill oil provided a lower serum TC level than fish oil, respectively, 46.1 ± 5.1 and 55.1 ± 6.9 mg/dl. Serum glucose levels of HFD groups were higher than those of C group, however only the difference between control and HFD group was found statistically significant (*p* = 0.01). Although mean serum insulin and HOMA-IR levels of HFD groups had a tendency to be higher compared to C group, no statistically significant difference was obtained (*p* = 0.13; *p* = 0.09, respectively). Serum leptin levels of HFD groups were higher than the control group, but only the difference between the control and the HFD group was significant (*p* = 0.03). The supplementation of marine oils did not result in a significant difference in serum leptin levels compared to either control or HFD groups (C *vs.* FO-HFD *p* = 0.80; C *vs.* KO-HFD *p* = 1.0; HFD *vs.* FO-HFD *p* = 0.62; HFD *vs.* KO-HFD *p* = 0.14). Also, no significant difference was found in serum levels of ghrelin among the groups (*p* = 0.29).

**Table 3:** Serum biochemical parameters of rats.

	C (*n* = 6)	HFD (*n* = 9)	FO-HFD (*n* = 9)	KO-HFD (*n* = 9)	p
TG (mg/dl)	65.5 ± 11.3	112.8 ±32.5^a^	93.8 ± 16.0	83.9 ±14.2^b^	**0.002**
TC (mg/dl)	43.2 ± 3.5	55.0 ±7.0^a^	55.1 ±6.9^a^	46.1 ±5.1^b,c^	**0.001**
HDL-C (mg/dl)	23.7 ± 2.0	22.4 ± 3.0	26.0 ± 3.3	22.4 ± 3.0	1.360
LDL-C (mg/dl)	17.0 ± 1.4	21.2 ±3.6^a^	18.9 ± 1.76	20.5 ± 2.8	**0.023**
Glucose (mg/dl)	97.8 ± 13.9	131.0 ±25.8^a^	121.8 ± 19.5	114.8 ± 19.5	**0.017**
Insulin (mIU/L)	8.0 ± 2.4	16.7 ± 10.1	13.8 ± 7.7	11.3 ± 4.3	0.131
Homa-IR	1.8 ± 0.4	5.7 ± 4.7	3.2 ± 1.2	4.2 ± 2.6	0.097
Leptin (pg/ml)	1773.8 ± 378.9	2724.3 ±832.5^a^	2255.1 ± 402.9	2055.2 ± 569.4	**0.027**
Ghrelin (ng/ml)	12.4 ± 1.6	10.2 ± 3.0	10.0 ± 2.4	10.4 ± 2.5	0.289

**Notes.**

Serum biochemical parameters of rats fed control (C, *n* = 6), high fat diet (HFD, *n* = 9), fish oil-high fat diet (FO-HFD, *n* = 9) or krill-oil high fat diet (KO-HFD, *n* = 9). Data is shown as mean ± standard deviation. ^a,b,c^Mean values with unlike superscript letters were significantly different (*p* < 0.05).

a C *vs.* HFD or C *vs.* FO-HFD or C *vs.* KO-HFD, *p* < 0.05.

b HFD *vs.* FO-HFD or HFD *vs.* KO-HFD, *p* < 0.05.

c FO-HFD *vs.* KO-HFD, *p* < 0.05. *p* was calculated by the One-way Anova test, and Bonferroni test was performed for post-hoc analysis.

**Figure 2:**
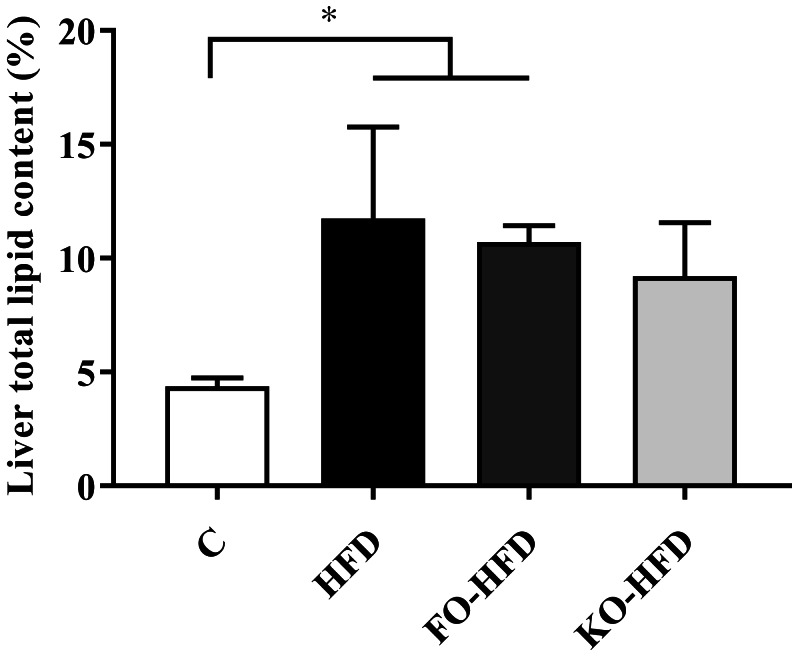
Liver total lipid content (%) in rats. Rats fed control (C, *n* = 6), high fat diet (HFD, *n* = 9), fish oil-high fat diet (FO-HFD, *n* = 9) or krill-oil high fat diet (KO-HFD, *n* = 9). Data is shown as mean ± standard deviation. ^∗^*p* < 0.05. *p* was calculated by the One-way Anova test, and Bonferroni test was performed for post-hoc analysis.

Fatty acid composition of rat liver samples is displayed at Table 4. Total SFA (*p* = 0.004) and PUFAs (*p* = 0.001) contents of livers were lower in HFD groups compared to the control group whereas MUFA content was higher in HFD groups (*p* = 0.01). In terms of individual fatty acids, the concentration of myristic acid (14:0, *p* = 0.01), pentadecylic acid (15:0, 0.017), palmitic acid (16:0, *p* = 0.020) and margaric acid (17:0, *p* = 0.005) in SFA category; palmitoleic acid (16:1, n-7 *p* = 0.001), oleic acid (18:1, n-9 *p* = 0.001) in MUFA category and linoleic acid (18:2, n-6 *p* = 0.001), arachidonic acid (20:4, n-6 *p* < 0.001) and docosahexaenoic acid (22:6, n-3 *p* = 0.006) in PUFAs category differed significantly among the groups. Total n-6 PUFAs content of liver samples was significantly lower in HFD groups than in the C group (*p* = 0.002), but no significant difference was observed among HFD groups (HFD *vs.* FO-HFD *p* = 0.680; HFD *vs.* KO-HFD *p* = 0.770; FO-HFD *vs.* KO-HFD *p* > 0.05. Total n-3 PUFAs content was significantly lower in HFD group compared to C group (*p* = 0.03), while it was similar to the C group in FO-HFD or KO-HFD groups (*p* = 0.430, *p* > 0.05, respectively). When EPA and DHA were considered individually, it was seen that the EPA (22:5, n-3) content of liver samples did not differ significantly among the groups (*p* = 0.178) whereas DHA (22:6, n-3) contents of the liver samples in HFD group was significantly lower compared to the C group (C *vs.* HFD *p* = 0.005). HFD reduced both n-3 and n-6 desaturase activity (*p* = 0.020, *p* < 0.001, respectively). In terms of n-6 desaturase activity, each HFD group had significantly lower activity levels compared to the C group (*p* < 0.001, for each); however, the lower n-3 desaturase activity was shown only in HFD and FO-HFD groups (C *vs.* HFD *p* = 0.030; C *vs.* FO-HFD *p* = 0.030; C *vs.* KO-HFD *p* = 0.130). Furthermore, the higher activity levels for both enzymes were recorded in KO-HFD group among HFD groups (Table 3). The mean of total lipid content in rat liver samples per group is compared at Fig. 2. Total lipid content of liver samples was significantly higher in HFD (11.7%) and FO-HFD (10.7%) groups than the C group (4.4%) (C *vs.* HFD *p* = 0.020; C *vs.* FO-HFD *p* = 0.040), whereas no significant difference was obtained between KO-HFD group (9.2%) and C group (C *vs.* KO-HFD *p* = 0.130). Among the HFD groups, KO-HFD group had the lowest total fat content, however, the difference was not deemed significant (HFD *vs.* FO-HFD *p* = 0.950; HFD *vs.* KO-HFD *p* = 0.580; FO-HFD *vs.* KO-HFD *p* = 0.860).

**Table 4:** Fatty acid composition of liver samples (%).

Fatty acids	C (*n* = 6)	HFD (*n* = 9)	FO-HFD (*n* = 9)	KO-HFD (*n* = 9)	p
**SFA**	42,7 ± 0,7	36,8 ± 2,1^a^	36,3 ± 0,6^a^	38,1 ± 2,1^a^	**0,004**
14:0	9,5 ± 0,8	6,1 ± 1,6^a^	6,4 ± 0,7^a^	6,4 ± 0,7^a^	**0,010**
15:0	0,6 ± 0,2	0,2 ± 0,0^a^	0,4 ± 0,2	0,2 ± 0,1^a^	**0,017**
16:0	16,1 ± 0,9	19,8 ± 1,2^a^	18,8 ± 0,8	18,7 ± 1,5	**0,020**
17:0	0,9 ± 0,2	0,3 ± 0,1^a^	0,3 ± 0,2^a^	0,4 ± 0,1^a^	**0,005**
18:0	14,9 ± 0,4	10,0 ± 2,1	10,1 ± 0,5	11,9 ± 3,1	0,440
22:0	0,8 ± 0,4	0,4 ± 0,1	0,2 ± 0,2	0,5 ± 0,4	0,199
**MUFA**	11,3 ± 1,5	30,0 ± 4,9^a^	23,3 ± 0,9^a^	22,5 ± 3,1^a^	**0,001**
16:1n-7	1,0 ± 0,2	0,6 ± 0,1^a^	0,4 ± 0,1^a^	0,4 ± 0,0^a^	**0,001**
18:1n-9	8,7 ± 1,0	24,9 ± 4,4^a^	21,3 ± 1,0^a^	20,6 ± 3,2^a^	**0,001**
24:1n-9	1,2 ± 0,7	1,1 ± 0,2	1,2 ± 0,3	1,2 ± 0,2	0,988
20:1n-9	0,4 ± 0,2	0,4 ± 0,3	0,5 ± 0,1	0,2 ± 0,1	0,453
**PUFA**	46,0 ± 1,4	36,2 ± 2,8^a^	40,5 ± 0,5^a^	39,5 ± 1,1^a^	**0,001**
**n-6 PUFA**	38,5 ± 1,3	31,5 ± 2,4^a^	33,6 ± 0,5^a^	33,5 ± 1,0^a^	**0,002**
18:2n-6	15,5 ± 0,8	19,7 ± 0,3^a^	20,8 ± 0,3^a^	20,1 ± 1,7^a^	**0,001**
20:2n-6	0,7 ± 0,3	0,5 ± 0,1	0,7 ± 0,2	0,6 ± 0,2	0,726
22:2n-6	0,9 ± 0,2	0,6 ± 0,1	0,9 ± 0,4	0,6 ± 0,2	0,217
20:4n-6	21,4 ± 0,8	10,7 ± 2,8^a^	11,2 ± 0,2^a^	12,2 ± 2,4^a^	**<0,001**
**n-3 PUFA**	7,4 ± 1,2	4,8 ± 0,6^a^	6,8 ± 0,9	6,0 ± 0,2	**0,022**
18:3n-3	0,7 ± 0,1	1,2 ± 0,5	1,5 ± 0,3	1,1 ± 0,3	0,073
20:3n-3	1,0 ± 0,2	0,6 ± 0,2	0,7 ± 0,3	0,7 ± 0,1	0,222
20:5n-3 (EPA)	0,6 ± 0,1	0,3 ± 0,2	0,4 ± 0,2	0,5 ± 0,2	0,178
22:5n-3	0,7 ± 0,3	0,7 ± 0,2	1,1 ± 0,4	0,9 ± 0,3	0,296
22:6n-3 (DHA)	4,5 ± 0,9	2,1 ± 0,4^a^	3,1 ± 0,4	2,9 ± 0,4	**0,006**
**n-6/n-3**	5,3 ± 1,0	6,6 ± 0,6	5,0 ± 0,8	5,6 ± 0,2	0,097
**n-3 desaturase activity**	0,9 ± 0,3	0,3 ± 0,2^a^	0,3 ± 0,1^a^	0,5 ± 0,2	**0,022**
**n-6 desaturase activity**	1,3 ± 0,1	0,5 ± 0,2^a^	0,5 ± 0,0^a^	0,6 ± 0,2^a^	**<0,001**

**Notes.**

Liver fatty acid composition of rats fed control (C, *n* = 6), high fat diet (HFD, *n* = 9), fish oil-high fat diet (FO-HFD, *n* = 9) or krill-oil high fat diet (KO-HFD, *n* = 9). SFA; saturated fatty acids, MUFA; monounsaturated fatty acids, PUFA; polyunsaturated fatty acids. Data is shown as mean ± standard deviation.

a Mean values with unlike superscript letters were significantly different (*p* < 0.05).

b C vs. HFD or C vs. FO-HFD or C vs. KO-HFD. *p* was calculated by the One-way Anova test, and Bonferroni test was performed for post-hoc analysis.

**Figure 3:**
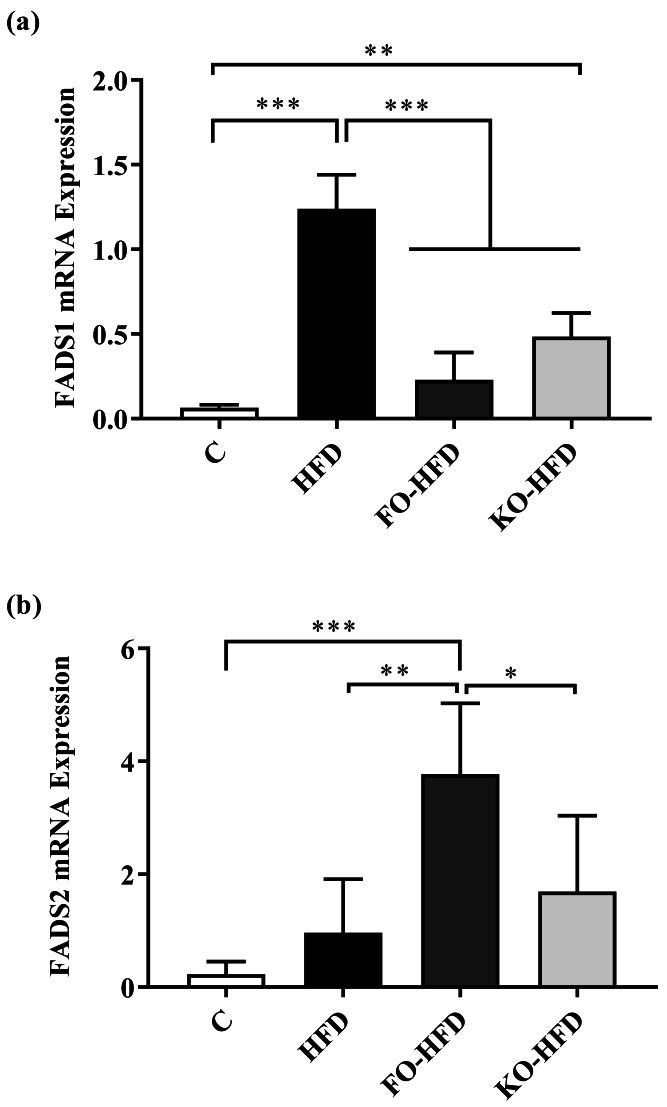
Fatty acid desaturase gene expression in rats. (A) FADS1 gene expression and (B) FADS2 gene expression in rats fed a control (C), high fat diet (HFD), fish oil-high fat diet (FO-HFD) or krill-oil high fat diet (KO-HFD). Rats fed control (C, *n* = 6), high fat diet (HFD, *n* = 9), fish oil-high fat diet (FO-HFD, *n* = 9) or krill-oil high fat diet (KO-HFD, *n* = 9). Data is shown as mean ± standard deviation. *p* was calculated by the one-way ANOVA test, and Bonferroni test was performed for post-hoc analysis. ^∗^*p* < 0.05; ^∗∗^ *p* < 0.01; ^∗∗∗^ *p* < 0.001.

Figures 3A and 3B show the gene expression levels of FADS1 and FADS2 in rat livers. HFD and KO-HFD groups had higher expression levels of FADS1 than the C group had (*p* < 0.001 and *p* = 0.010, respectively). Among the HFD groups, the expression level of FADS1 was decreased significantly by the LC n-3 PUFA supplementation (*p* < 0.001 for each), and this effect was more pronounced by the supplementation of fish oil compared to krill oil (*p* = 0.840). Furthermore, the high fat diets showed an opposite regulation for FADS2 gene expression. The expression of FADS2 gene was significantly higher in FO-HFD group compared to the C group (*p* < 0.001), HFD group (*p* = 0.001) and KO-HFD group (*p* = 0.01). The supplementation of fish oil down-regulated FADS1 gene expression whereas up-regulated FADS2 gene expression. HFD and KO-HFD also showed a tendency to increase the expression level of FADS2 compared to control diet (C *vs.* HFD *p* = 1.000; C *vs.* KO-HFD *p* = 0.190).

## Discussion

LC n-3 PUFAs has been under examination in terms of its potential anti-obesity activity in the last decades (Bender et al., 2014; Buckley & Howe, 2010; Du et al., 2015; Simopoulos, 2016). In addition to the contradictory findings that makes it difficult to develop a strategy for the supplementation of LC n-3 PUFAs, the source of LC n-3 PUFAs has also been questioned regarding its efficiency (Ulven & Holven, 2015). This study has investigated the effects of fish oil and krill oil supplementation on weight gain, dietary intake, biochemical parameters related to obesity, liver fatty acid composition, and desaturase gene expressions in a rat model; and it was shown that high-fat diets supplemented with fish oil and krill oil affect some of these parameters differently.

Although there have been various LC n-3 PUFAs recommendations by authorities for chronic conditions such as cardiovascular disease, hyperlipidemia and hypertension associated with obesity, there is no recommendation on LC n-3 PUFAs supplementation for the prevention or the treatment of obesity (Lorente-Cebrián et al., 2013). The main explanation for this fact is the lack of consistency in the findings of studies that have examined the effects of LC n-3 PUFAs on body weight and appetite (Bertrand et al., 2013; Pérez-Matute et al., 2007; Philp et al., 2015; Rossmeisl et al., 2012; White et al., 2010). For instance, Ferramosca et al. demonstrated that rats fed high-fat diet supplemented with 2.5% krill oil gained less weight than the rats that are fed only high-fat diet (Ferramosca et al., 2012).Whereas, Tandy et al. (2009) reported that the consumption of high-fat diet containing different amounts of krill oil (0%, 1.25%, 2.5%, 5.0%) for 8 weeks caused a higher weight gain but less feed consumption compared to the standard diet group, without any difference among krill oil groups in a rat model. Similarly, this study showed that high-fat diets lead to higher weight gain even though less feed is consumed. Therefore, this study supported the hypothesis that high-fat diets provide obesogenic effects not only by increasing food consumption, but also through other mechanisms, such as altering fat oxidation ratio, modifying uncoupling protein 1 gene expression, reducing thermogenesis, increasing lipoprotein lipase activity, and causing insulin resistance (Fromme & Klingenspor, 2011; Hariri & Thibault, 2010). Furthermore, according to this study, LC n-3 PUFAs supplementation either from fish oil or krill oil could not prevent weight gain induced by a high-fat diet. However, their anti-obesity effects might be more apparent with a low-fat diet (Poudyal et al., 2013), suggesting minimal effects of LC n-3 PUFAs on managing body weight. Also, it was noted that the ratio of LC n-3 PUFAs to total dietary fatty acids might be critical in their metabolic efficiency on obesity (Poudyal et al., 2013). Therefore, supplementation of LC n-3 PUFAs in different ratios and in diets with low-to-normal fat contents might provide a better understanding of their effects on weight gain. There is limited evidence on the effects of LC n-3 PUFAs supplementation on leptin and ghrelin levels. A meta-analysis demonstrated that while LC n-3 PUFAs supplementation increased serum leptin levels in obese individuals, they decreased in non-obese individuals (Hariri & Thibault, 2010). However, inconsistent results have been reported in different studies (Gray et al., 2013; Pérez-Matute et al., 2007; Puglisi, Hasty & Saraswathi, 2011). For instance, in a study conducted by Huerta et al. (2015), 1.3 g/day EPA supplementation in overweight or obese women for 10 weeks resulted in a lower decline in serum leptin levels, but not significant changes in serum ghrelin levels (Huerta et al., 2015). Among the animal studies, Pérez-Matute et al. (2007) showed that EPA supplementation to cafeteria diet resulted in an increase in leptin levels whereas serum leptin levels decreased when EPA was supplemented with a standard control diet, suggesting an interaction between diet composition and EPA supplementation on serum leptin levels (Pérez-Matute et al., 2007). Furthermore, the supplementation of n-3 LCPUFAs decreased serum leptin levels caused by high-fat diet in a nonalcoholic fatty liver disease (NAFLD) model (Ding & Wang, 2014). To our knowledge, the present study is the first to reveal the effects of krill oil given along with a high-fat diet on serum leptin and ghrelin levels. The high-fat diet resulted in the highest serum leptin levels while both LC n-3 PUFAs sources suppressed the effect of high-fat diet on serum leptin level. This might be a protective mechanism for leptin resistance, which is common in obese individuals. Additionally, none of the high-fat diets produced any significant alterations in serum ghrelin levels. This might be explained by the different duration of intervention in different studies. It is clear that long intervention periods might provide apparent effects on serum leptin or ghrelin levels.

Studies that have examined the effects of LC n-3 PUFAs supplementation on lipid profile and glycemic response have also reported conflicting results (Ivanova et al., 2015; Vigerust et al., 2013; Yang et al., 2016). Yang et al. (2016) showed that a high-fat diet with a 40% of fat content and 2 g/100 g krill oil did not change fasting glucose, TC or HDL-C levels but reduced TG and LDL-C levels in a ten-week intervention (Yang et al., 2016). In a study investigating the effect of krill oil and fish oil containing the same amount of EPA+DHA added to a high-fat diet on lipid metabolism, krill oil decreased serum TG and TC levels without affecting serum LDL-C and HDL-C levels whereas, fish oil decreased serum TC and LDL-C levels (Vigerust et al., 2013). In the current study, krill oil significantly decreased serum TG and TC levels but did not affect serum HDL-C or LDL-C levels, while fish oil did not affect any component of the lipid profile. In terms of glucose homeostasis, it was shown that LC n-3 PUFAs supplementation did not improve insulin resistance caused by a high-fat diet. LC n-3 PUFAs demonstrate their effects on glucose and lipid metabolism through the similar mechanisms such as modulating the levels of genes and enzymes involved in lipogenesis and beta oxidation in the skeletal muscle and liver, activating the peroxisome proliferator-activated receptor-α (PPARα), reducing the level of inflammatory biomarkers including tumor necrosis factor-α (TNF-α), and increasing adiponectin and AMPK levels (Bjørndal et al., 2012; Ivanova et al., 2015; Neschen et al., 2007; Pérez-Matute et al., 2007; Vigerust et al., 2013; Yang et al., 2016).

Regarding the effects of LC n-3 PUFAs supplementation on liver total lipid and fatty acid composition, the high-fat diet increased the total lipid content of liver and changed its fatty acid composition, however, the fish oil could not alter the effects induced by high-fat diet. For the control diet group of this study, krill oil had similar effects in terms of total lipid content. In other words, since the total liver lipid ratio of the group given krill oil is similar to the control group, krill oil may have a protective effect on fatty liver diseases, but further studies are needed. The increase in total lipid content of liver by high-fat diet and protective effect of krill oil on this parameter were parallel with the findings of previous studies (Burri et al., 2011; Ralston et al., 2015; Tillander et al., 2014; Zhang et al., 2019). Burri et al. (2011) reported that krill oil reduced hepatic lipogenesis by regulating many metabolic pathways such as lipogenic enzyme activities, gene expression, and mitochondrial respiratory chain better than fish oil (Burri et al., 2011). Moreover, it was noted that krill oil could reduce fat accumulation in the liver by reducing Acetyl-CoA carboxylase and fatty acid synthase. Although the EPA and DHA contents of krill oil are lower than that of fish oil, krill oil can provide a similar EPA and DHA content in the liver as fish oil(Tillander et al., 2014). However alterations in some of the fatty acids were contradictory with the earlier findings, probably due to the variation in the amount of fish or krill oils or EPA used in the intervention, the duration of the intervention, and the animal models used in each study (Moreno-Aliaga, Lorente-Cebrián & Martínez, 2010). Previous studies reported that PUFAs intake decreases desaturase activity (Arbo et al., 2011; Cho, Nakamura & Clarke, 1999a; Cho, Nakamura & Clarke, 1999b). In this study, krill oil exhibited similar effects on total n-3 fatty acid desaturase activity to control diet, while high-fat or FO-HFD caused significant decrease in enzyme activity. This effect was explained by the phospholipid, fatty acid, and astaxanthin content of krill oil (Jia et al., 2012; Ni et al., 2015; Tandy et al., 2009).

It has been shown in cell cultures, baboons, mice, and humans that FADS1 and FADS2 gene expressions encoding desaturase enzymes can be controlled by the uptake of LC n-3 PUFAs (Chisaguano et al., 2013; Reardon et al., 2013; Su et al., 2016). In this study, high-fat diet increased FADS1 gene expression compared to control diet, however, both fish oil and krill oil diminished this increase, even though the expression levels caused by krill oil were still higher than that of the standard diet. The decreased FADS1 gene expression level by the supplementation of LC n-3 PUFAs was reported in the previous studies (Ralston et al., 2015; Reardon et al., 2013; Zhang et al., 2019). On the contrary, these studies also showed that LC n-3 PUFAs diminished the increase in the expression level of FADS2 gene caused by high-fat diet while this study observed the LC n-3 PUFAs enhanced the effect of high-fat diet on FADS2 gene expression. This might be explained by the compensatory mechanism of low expression levels in FADS1 gene that resulted in the increase in FADS2 gene expression. Moreover, the amount of LC n-3 PUFAs was different in this study compared to previous studies, with a doubled dosage in previous studies (Zhang et al., 2019).

The decreased FADS1 gene expression caused by the dietary LC n-3 PUFAs supplementation could be explained *via* a potential mechanism developed by the organism to protect the unsaturation index of cell membranes (Reardon et al., 2013). This study suggested that fish oil and krill oil can affect the expression levels of FADS1 and FADS2 genes differently, including a higher expression level for FADS1 gene by krill oil and for FADS2 gene by fish oil. To our knowledge, this is the first study in the literature that has shown the effects of krill oil on FADS1 and FADS2 gene expressions. The difference in the expression levels by source of LC n-3 PUFAs could be explained by the varied EPA and DHA content in these sources. The low levels of total n-3 and n-6 fatty acid activities in high-fat diet groups in comparison with the control group, despite the increase in desaturase gene expression indicates that factors other than gene expression may affect enzyme activity. It is known that enzyme activities are affected by physiological status, oxidative stress, hormones and other nutrients (Araya et al., 2004; Wang et al., 2006). In addition, the fatty acids formed by D5D and D6D enzyme activities are highly bioactive molecules, and their fatty acid ratios can be affected by many metabolic conditions (Sjögren et al., 2008).

The major limitation of this study was the fact that though the equal amounts of fish oil and krill oil were added to the feeds, they did not contain equal amounts of EPA and DHA. However, the supplemented amounts of the fish oil and krill oil were in parallel with the previous studies that simulated the dietary intakes in humans (Ferramosca, Conte & Zara, 2012; Tandy et al., 2009). Therefore, further studies with krill oil and fish oil that contain equal amounts of EPA and DHA are required to better understand their efficiency. Despite the limitation, this study made a significant contribution to the literature since, to our knowledge, it was the first study to examine the effects of krill oil on appetite hormones and desaturase gene expression.

## Conclusion

In conclusion, this study supported the moderate effects of krill oil on lipid profile, especially on TG and TC levels, however, failed to exhibit any significant contributions on glycemic response, weight management or obesity related hormones even through decreased the feed intake. Furthermore, it has shown that different sources of LC n-3 PUFAs might regulate lipid metabolism and expression of desaturase genes differently in rats. However, a better understanding of molecular mechanisms is required. Therefore, further studies, especially randomized controlled trials, are essential to determine the efficacy of different LC n-3 PUFAs sources in the treatment and prevention of obesity and obesity related diseases in humans.

## Supplemental Information

10.7717/peerj.12009/supp-1 Supplemental Information 1 Raw data

10.7717/peerj.12009/supp-2 Supplemental Information 2 Detailed methods

10.7717/peerj.12009/supp-3 Supplemental Information 3 Author checklist

## Additional Information and Declarations

### Data Availability

The following information was supplied regarding data availability: Raw data is available in the Supplemental Files.
