# Supplementary material for: Efficacy of krill oil versus fish oil on obesity-related parameters and lipid gene expression in rats: randomized controlled study
Source: PeerJ. 2021 Sep 27;9:e12009. doi: 10.7717/peerj.12009 (PMC8483003; doi:10.7717/peerj.12009)
Supplement: Supplemental Information 2 [file peerj-09-12009-s002.docx]

**ELİSA**

In this study, specific kits for each protein suitable for rat serum were used for the analysis of serum insulin, leptin and ghrelin parameters. Determination of insulin (Sunredbio, Shanghai-catalog no: 201-11-0708), leptin (Sunredbio, Shanghai-catalogue no: 201-11-0456) and ghrelin (Sunredbio, Shanghai-catalogue no: 201-1650) levels of protein -The Enzyme Associated Immune Assay (ELISA) method, which is based on antibody binding, was carried out by applying the kit protocol in Erzurum Atatürk University Faculty of Medicine, Department of Pharmacology. Reagents, samples and standards were primarily prepared according to the kit protocol. Then, standards and samples were placed in the specified amounts as one empty well into the 96-well plate. It was studied with the duplication method. It was incubated at 37^o^C for one hour. Chromogen solutions were added by washing five times in an automatic washing machine (Stat Fax 2600). The reaction was kept at 37^o^C for 10 minutes. The amount of protein was determined in a spectrophotometer (Biotek, Epoch-nano drop apparatus) device by adding the stop solution within 10 minutes.

**LIPID COMPOSİTİON**

1. **Determination of the amount of lipid extracted from samples**

In order to extract total lipid from the samples, ~1 g samples were transferred to 70 ml glass tubes and 20 ml of a chloroform/methanol (2:1 v/v) mixture containing 0.01% (w/v) butylated hydroxytoluene was added to them and 1 minute with ultrathorax. After fragmentation, it was filtered using Whatman No: 1 filter paper by means of a vacuum pump. The filtered samples were transferred to separate clean and dry glass tubes and 2% of the samples were added with MgCl26H2O (4 ml for each 20 ml solution). The tubes were filled with nitrogen for 1 minute and tightly closed so that no air leaks, then vortexed for 1 minute and stored at room temperature and in the dark for one day for phase formation.

At the end of the 24-hour period, the sub-phase formed was transferred to a clean and dry tube with a pasteur pipette. The tubes, in which some chloroform was added, were placed in the nitrogen evaporator system and subjected to heating and nitrogen gas. After evaporation of chloroform and solvents, the remaining samples were transferred to 10 ml glass test tubes, which were tared and recorded and evaporation continued. After the solvents had completely evaporated, the tubes were weighed at regular intervals and recorded. The weights were continued until the weights were stabilized and the amount of lipids in the tubes was calculated by gravimetric method. A little chloroform was added to the glass tubes and nitrogen gas was filled into them, the lids were tightly closed and they were brought to -20 °C (Folch et al. 1957).

1. **Preparation of fatty acid methyl esters (FAMEs)**

1.5 ml of 2 M NaOH solution was added to the glass test tubes containing the lipids obtained from the samples, and the lids were tightly closed under nitrogen. After filling the tubes with nitrogen gas, the caps were tightly closed and the lipids were kept in an oven (Binder FD 53) heated to 80°C for 1 hour to saponify. At the end of the 1-hour period, 2 ml of 14% BF3 (Borontriflouride methanol) was added to the samples cooled at room temperature, and nitrogen gas was filled again and kept at 80°C for another half hour. At the end of the period, the samples were taken back to room temperature and allowed to cool. 1 ml of hexane was added to the cooled samples and vortexed. Then 1 ml of ultrapure water was added to the tubes and vortexed again. Finally, hexane was added to the tubes once more and the upper phase was taken with a pasteur pipette and transferred to new glass test tubes containing sodium sulfate (Na_2_SO_4_). The collected hexane layer was transferred to 2 ml GC vials, filled with nitrogen gas and capped (Metcalfe and Schmitz 1961). The prepared vials were placed in a gas chromatography (GC) device to detect fatty acids.

1. **Determination of fatty acids**

The FAME obtained were separated using gas chromatography (Agilent 6890 N, Santa Clara, USA) equipped with a flame ionization detector, a 100 m capillary column (CP-Sil 88 Agilent 6890 N, Santa Clara, USA) and an auto injector. The carrier gas was helium at a flow rate of 35 mL/min. The initial temperature of the oven was 165 °C which was maintained for 15 min, and then there was an increase to 200 °C by increments of 5 °C/min, and subsequently there was maintenance at 200 °C for 47 min. Fatty acids were then identified by comparing the retention times to those of a standard mixture of fatty acids (Sigma-Aldrich, Steinhein, Germany).
